# ﻿Re-discovery and taxonomic clarification of *Oreocharisleveilleana* (Gesneriaceae) in Guizhou, China, over 100 years

**DOI:** 10.3897/phytokeys.242.115955

**Published:** 2024-05-10

**Authors:** Xiao-Kai Xiong, Song-Tao He, Yu-Lu Zhou, Fang Wen, Xin-Xiang Bai

**Affiliations:** 1 College of Forestry, Guizhou University, CN-550025, Guiyang, Guizhou, China Guizhou University Guiyang China; 2 Guangxi Key Laboratory of Plant Conservation and Restoration Ecology in Karst Terrain, Guangxi Institute of Botany, Guangxi Zhuang Autonomous Region and Chinese Academy of Sciences, CN-541006, Guilin, Guangxi Zhuang Autonomous Region, China Guangxi Institute of Botany, Guangxi Zhuang Autonomous Region and Chinese Academy of Sciences Guilin China; 3 Gesneriad Committee of China Wild Plant Conservation Association, National Gesneriaceae Germplasm Resources Bank of GXIB, Gesneriad Conservation Center of China (GCCC), CN-541006, Guilin, Guangxi Zhuang Autonomous Region, China National Gesneriaceae Germplasm Resources Bank of GXIB, Gesneriad Conservation Center of China (GCCC) Guilin China

**Keywords:** *Oreocharis*, *Petrocodon*, *Petrocodoncoccineus*, new combination, synonym

## Abstract

*Oreocharisleveilleana* Fedde was collected in Ta-pin in 1910 and published in 1911. The collected location was verified within western Luodian County, Guizhou Province, China. However, there have been no records of the species’ collection for more than 100 years since then. After extensive investigations by our research team on the type locality and its surrounding areas, we found that it is widely distributed in western Luodian County and eastern Wangmo County, Guizhou Province, China. During further research on the original literature, type specimens and type locality of *O.leveilleana*, the taxonomic position of *O.leveilleana*, which was once treated as a synonym of *O.auricula* (S.Moore) C.B.Clarke, was found to have a taxonomic problem. Through morphological research combined with geographical distribution analysis, it has been determined that it should belong to the genus *Petrocodon* Hance and it is the same species as *P.coccineus* (C.Y.Wu ex H.W.Li) Yin Z.Wang. According to the regulations and suggestions of the 2018 "International Code of Nomenclature for Algae, Fungi, and Plants (Shenzhen Code)", we propose and confirm a new combination – *Petrocodonleveilleanus* (Fedde) X.X.Bai & F.Wen and treat *P.coccineus* as a synonym of the new combination. Due to its unique bright red flowers within *Petrocodon*, its original Chinese name has been retained.

## ﻿Introduction

The genus *Oreocharis* Benth., established by G. Bentham (1876), belongs to the tribe Trichosporeae, subfam. Didymocarpoideae, Gesneriaceae (Weber et al. 2013). Currently, there are more than 150 recorded species (excluding infraspecific taxa), while China has about 158 taxa (including 15 varieties). These plants are predominantly distributed in the south-western region of China and most parts of southern China, with about ten species distributed in Vietnam, Myanmar, Japan and Thailand (GRC 2023). *Oreocharis* is a diverse group of plants with various morphologies. There have been significant discrepancies in taxonomic viewpoints amongst scholars in different periods, leading to substantial changes in its systematic position. It was not until Möller et al. (2011), based on molecular evidence and morphological analysis, merged nine genera including *Ancylostemon* Craib, *Opithandra* B.L.Burtt, *Isometrum* Craib, *Tremacron* Craib, *Bournea* Oliv., *Dayaoshania* W.T.Wang, *Thamnocharis* W.T.Wang, *Deinocheilos* W.T.Wang, *Paraisometrum* W.T.Wang and the rosulate taxa of *Briggsia* Craib into the enlarged concept of *Oreocharis*, that the systematic revision of this genus was essentially completed. Although the genus *Bournea* was considered an independent genus again (Chen et al. 2020), further research did not support this view (Lv et al. 2022). The frequent and extensive revisions in taxonomy have led to changes in the systematic positions and scientific names of numerous species, making the study of *Oreocharis* even more complex (Fu et al. 2019).

Additionally, some early published species were established, based on only one or two specimens, with limited or ambiguous information on their type localities. Furthermore, these species often lack important and valuable taxonomic characteristics and there have been no further collection records or literature after publication. The names and habitats of the type locality of some species have also changed over time, making it difficult to rediscover them. These issues have posed challenges for subsequent taxonomic research. For example, *Oreocharisrhytidophylla* C.Y.Wu ex H.W.Li was considered a doubtful species due to the lack of descriptive information regarding its floral characteristics. It was not until recently that Zhang et al. (2019) collected flowering specimens from its type locality, confirming it as a natural species rather than a doubtful one.

*Oreocharisleveilleana* Fedde, first collected in 1910 and described as a new species under the genus *Oreocharis* in 1911, had not been recorded again for over 100 years. Due to limited specimen and literature records, it was considered a doubtful species (Pan 1987; Wang et al. 1990). Subsequently, it was merged with *O.auricula* (S.Moore) C.B.Clarke due to the temporary unavailability of additional literature and collection records (Wang et al. 1998). Further, due to the unavailability of molecular data for this species, in the study by Möller et al. (2011), *O.leveilleana* was still treated as a synonym of *O.auricula*.

In recent years, our research team conducted a comprehensive survey of the Gesneriaceae in Guizhou Province. In April 2023, during the surveys in Luodian County and Wangmo County, we discovered an interesting species of Gesneriaceae. We observed the living plants and recorded their morphological characteristics. We were very certain that the vegetative organs of this species are morphologically almost identical to the type specimen of *O.leveilleana* (E00067459). To ensure that our judgement was not mistaken, we confirmed it was *O.leveilleana* after carefully comparing it with the descriptions in literature and the type specimen. Furthermore, while examining the literature on species in the genus *Oreocharis*, we found some issues with the current taxonomic position of *O.leveilleana*. We believe that *O.leveilleana* should be classified under *Petrocodon* Hance and that it is the same as *P.coccineus* (C.Y.Wu ex H.W.Li) Yin Z.Wang. *P.coccineus* was first published as a new species in 1982 as the type species of *Calcareoboea* C.Y.Wu ex H.W.Li, which was subsequently transferred to *Petrocodon*, based on molecular evidence and it has since become a species under *Petrocodon* (Wang et al. 2011).

## ﻿Materials and methods

We reviewed the original literature and related records of *Oreocharisleveilleana* and *Petrocodoncoccineus*, including the original literature description, information on type specimens and geographical distribution. In addition, we accessed the digital plant specimens of the E, K, P, IBK, PE, KUN and GXMI collections to check the type specimens and high-resolution images of both species, along with other specimens. The primary sources of original literature are from BHL (www.biodiversitylibrary.org), Tropicos (http://www.tropicos.org), and IPNI (http://www.ipni.org). Additionally, we made multiple visits to the type localities of both species to conduct extensive field surveys and document the growth and distribution of populations. The study was carried out using classical plant taxonomic methods.

## ﻿Results and discussion

### ﻿Comparison and discussion of morphological characteristics

*Oreocharisleveilleana* was originally published by French botanist, Augustin Abel Hector Léveillé (1911a). It was collected as a specimen with the number *2051* (Holotype E, E00067459) from Ta-pin (now located in the western Luodian County, Guizhou Province, China) by Joseph Henri Esquriol in 1910. The new species was described and published under the genus *Oreocharis*, named *O.esquirolii* H.Lév. as a tribute to the collector’s name on the specimen. In the same year, another botanist Friedrich Karl Georg Fedde (1911) realised that this scientific name was identical to another species, *O.esquirolii* H.Lév., also published by H. Léveillé (1911b). To avoid confusion in taxonomy, a replacement name was proposed. The specific epithet was changed to “*leveilleana*”, derived from the Latinised version of the original from Augustin Abel Hector Léveillé. According to the protologue, the morphological characteristics of *O.leveilleana* are simply described as follows: leaf blades oblong, 4–7 cm in length and 1.5–2 cm in width; margin sparsely serrulate, nerves parallel; petioles 3–6 cm long, dark brown tomentum; capsule ca. 5 cm long.

As is recorded in *Flora Reipublicae Popularis Sinicae* (Vol. 69) “based on the type specimen photos, this plant is glabrous; does this species belong to the genus *Oreocharis*? Whether this species belongs to the genus *Oreocharis* should be determined when complete specimens will be collected”. Therefore, Wang et al. (1990) classified this species as a doubtful species. Later, *O.leveilleana* was treated as a synonym of *O.auricula* (Wang et al. 1998), although it was also treated as a synonym of *O.sericea* H.Lév. in related monographs (Li and Wang 2005; Wei et al. 2010). Ultimately, scholars followed the taxonomic viewpoint in *Flora of China* (Vol. 18) (Möller et al. 2011).

**Figure 1.**
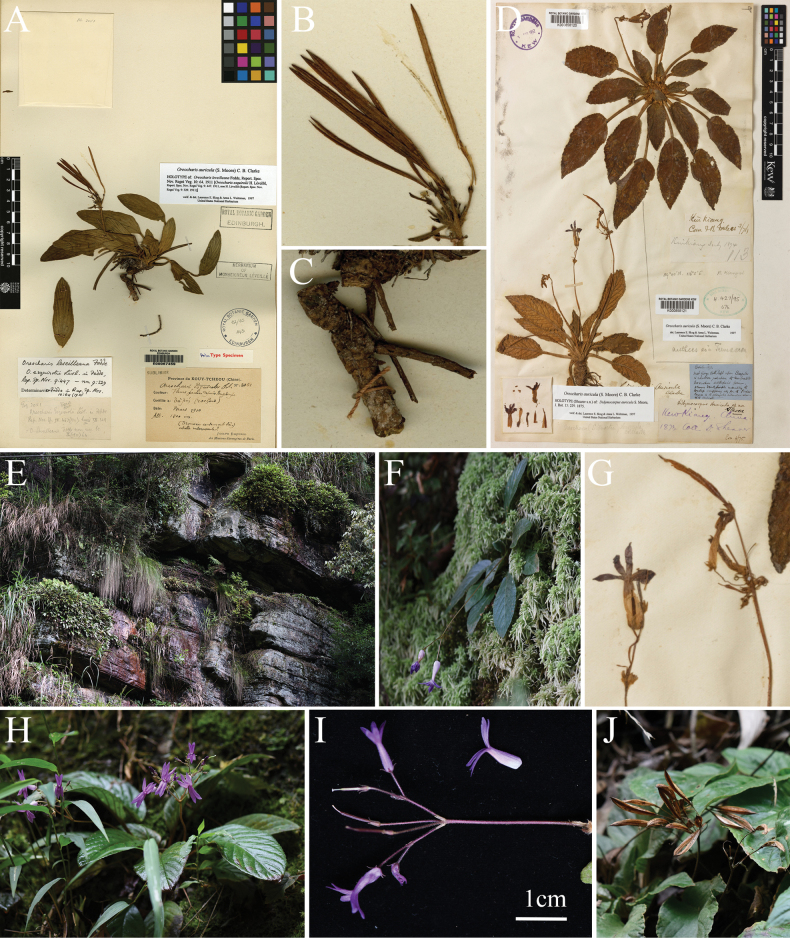
Morphological comparisons of *Oreocharisleveilleana* and *O.auricula***A–C***O.leveilleana***D–J***O.auricula***A** holotype (E00067459) **B** capsules and bracts **C** rhizome **D** holotype (K000858120) **E** habitat **F, H** habit **G, I** inflorescence **J** capsules (Xin-Xiang Bai took the field survey photographs).

Careful observation of specimen *2051* (Holotype E, E00067459) (Fig. 1A) showed the following morphological characteristics of this specimen: rhizome robust, terete, ca. 6 mm in diameter; petiole densely puberulent; leaf blades densely pubescent, margin denticulate-serrulate; peduncle robust, densely puberulent, terete, 2–3 mm in diameter, unbranched; pedicel short, 2–6 mm long; inflorescence subumbellate, bracts more than 5, dense, densely pubescent; capsule linear, loculicidally dehiscent into two valves. Detailed morphological comparisons with *O.auricula* are provided in Fig. 1. Moreover, the morphological characteristics shown by specimen *2051* are clearly distinguished from those of other species within *Oreocharis*, such as inflorescence subumbellate (vs. cymes), bracts more than 5, dense (vs. bracts 2 or 3, opposite or whorled), capsule linear and loculicidally dehiscent into two valves (vs. capsule lanceolate-oblong or oblong). However, these morphological characteristics match well with *Petrocodoncoccineus*.

**Figure 2.**
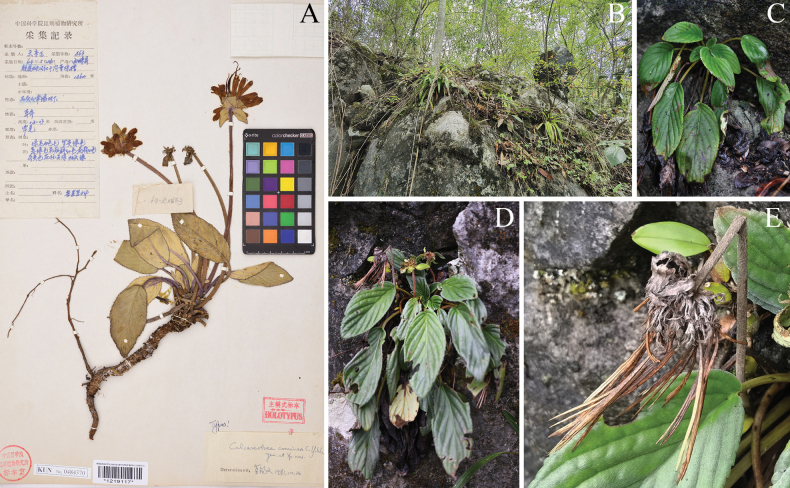
*Petrocodoncoccineus***A** holotype (KUN1219117) **B** habitat **C** habit **D** plant with infructescence **E** infructescence and peduncle (Xin-Xiang Bai took the field survey photographs).

*Calcareoboeacoccinea* C.Y.Wu ex H.W.Li was published, based on the specimen of *S.C.Wang 463* (Holotype KUN, KUN1219117) (Fig. 2A) collected from Xichou County, Yunnan Province, China (Li 1982). The scientific name is derived from its bright red corolla and the genus *Calcareoboea* was established with *C.coccinea* as the type species. It was once a monotypic genus. Subsequently, Wang et al. (2011) provided molecular evidence to transfer the genus *Calcareoboea* into the genus *Petrocodon*. Consequently, the scientific name for the species was changed to *Petrocodoncoccineus* (C.Y.Wu ex H.W.Li) Yin Z.Wang. The characteristics of the *P.coccineus* are as follows: inflorescence subumbellate and scapiform; peduncle elongated and robust, terete with numerous leafy involucres at the apex; bracts 6–10 or more, dense, small, linear; flowers with short pedicel, 2–4 mm long; capsule linear, ca. 6 cm long with stalk, loculicidally dehiscent into two valves. These morphological features are consistent with those of *Oreocharisleveilleana*.

### ﻿Locality research

By consulting relevant historical records and referring to the Gazetteers of China History Collections (https://www.cvh.ac.cn/topics/counties.php), it has been found that the type locality of *Oreocharisleveilleana*, Ta-pin, is in western Luodian County, Guizhou Province. Further extensive investigations were conducted in the type locality and surrounding areas, revealing that *Petrocodoncoccineus* is widely distributed in the western Luodian County and the eastern Wangmo County, Guizhou Province (Fig. 2).

In conclusion, based on the study of morphology, combined with geographical distribution analysis, according to the latest taxonomic viewpoint of the Gesneriaceae, *Oreocharisleveilleana* should belong to the genus *Petrocodon* and it is actually the same species as *P.coccineus*. Given that the publication of *Oreocharisleveilleana* predates the one of *Petrocodoncoccineus* and, following the regulations and suggestions of the 2018 "International Code of Nomenclature for Algae, Fungi, and Plants (Shenzhen Code)" (Turland et al. 2018), this study proposes a new combination – *Petrocodonleveilleanus* (Fedde) X.X.Bai & F.Wen and treats *P.coccineus* as the synonym of the new combination. The Chinese name of *P.leveilleanus* (= *P.coccineus*) is retained because it more accurately expresses the morphological characteristics of this species and is widely accepted.

### ﻿Taxonomic treatment

#### ﻿ Petrocodon leveilleanus

Taxon classificationPlantaeLamialesGesneriaceae

(Fedde) X.X.Bai & F.Wen comb. nov.

6CC09ACA-9D77-541D-A6A8-51B428D0D57B

urn:lsid:ipni.org:names:77341571-1

Fig. 3

Oreocharis leveilleana Fedde, Repert. Spec. Nov. Regni Veg. 10: 64. 1911. – O.esquirolii H.Lév., Repert. Spec. Nov. Regni Veg. Berlin. 9: 447. 1911 [non Léveillé (1911b: 329)]. Type: China, Guizhou: Ta-pin, March 1910, *Joseph Henri Esquriol 2051* (E: holotype, E00067459).
= Petrocodoncoccineus (C.Y.Wu ex H.W.Li) Yin Z.Wang, J. Syst. Evol. 49(1): 60. 2011. syn. nov. – Calcareoboeacoccinea C.Y.Wu ex H.W.Li, Acta Bot. Yunnan. 4(3): 243. 1982. Type: China, Yunnan: Xichou, Gankou, 26 May 1964, *C.S. Wang 463* (KUN: holotype, KUN1219117).

##### Chinese Vernacular name.

朱红苣苔 (Zhū Hóng Jù Tái).

##### Distribution and habitat.

Luodian County, Wangmo County, Libo County and Zhenning County of Guizhou Province, as well as in the southeastern Yunnan Province and the southwestern and northern Guangxi Zhuang Autonomous Region of China and northern Vietnam. The species grow on wet rocks under the forest canopy at an elevation of 500–1500 m.

**Figure 3.**
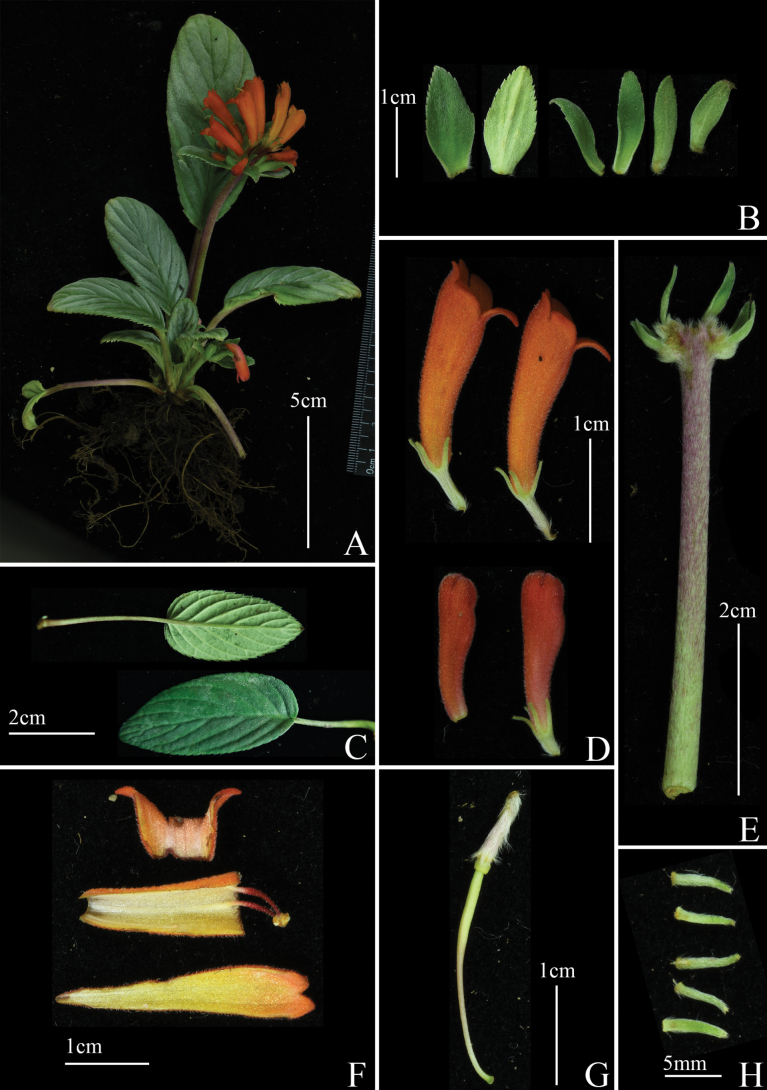
*Petrocodonleveilleanus***A** plant **B** bracts **C** leaves **D** corolla **E** peduncle **F** dissected flower **G** Pistil **H** Sepals (photographs by Xin-Xiang Bai).

##### Specimens examined.

**China. Guangxi Zhuang Autonomous Region**: Napo County, 1220 m elev., 2 Jun 2006, Haining Qin et al. 602018 (PE); Napo County, 1100 m elev., 8 Nov 1992, Yinzheng Wang 92038 (PE); Napo County, 1220 m elev., 24 Apr 1981, Ding Fang et al. 22421 (PE); Jingxi City, elevation unknown, 28 May 1990, Xiuxiang Chen et al. 14769 (GXMI); Du’an County, 274 m elev., 19 Jul 2019, Bo Pan et al. GXIBPB057B01 (KUN). **Yunnan Province**: Malipo County, 1000 m elev., 3 Jan 1940, Qiwu Wang 86147 (KUN); Malipo County, 1400–1500 m elev., 9 Dec 1992, Yinzheng Wang 92101 (PE); Malipo County, 1300–1500 m elev., 2 Nov 1947, Guomei Feng 13508 (KUN); Funing County, 1000 m elev., 8 May 1940, Qiwu Wang 87144 (KUN); Funing County, 1400 m elev., 18 Sep 2006, Lianming Gao GLM-06184 (KUN); Xichou County, elevation unknown, 7 May 1959, Quan’an Wu 7961 (KUN). **Vietnam. Son La Province**: Moc Chan District, 1400–1500 m elev., 6 Mar 2001, D.K.Harder et al. 7342 (E). **Hoa Binh Province**: Mai Chau District, 1000 m elev., 20 Oct 2002, Northern Vietnam First Darwin Expedition 3 (E); Mai Chau District, 1066 m elev., 26 Sep 2018, S. Razafimandimbison et al. 2373 (P). **Ha Giang Province**: Vi Xuyen District, 300–1050 m elev., 16 Feb 2001, D.K.Harder 6449 (E); Quang Ba District, 1100 m elev., 3 Apr 2000, D.K.Harder et al. 4946 (E).

## Supplementary Material

XML Treatment for Petrocodon leveilleanus

## ﻿Additional information

### Author ORCIDs

Fang Wen https://orcid.org/0000-0002-3889-8835

Xin-Xiang Bai https://orcid.org/0000-0003-2449-6664

### Data availability

All of the data that support the findings of this study are available in the main text.

## References

[B1] Bentham G (1876) Gesneriaceae. In: Bentham G Hooker JD (Eds) Genera Plantarum 2(2).Lovell Reeve & Co., London, 990–1025.

[B2] Chen WH Zhang YM Guo SW Zhang ZR Shui YM (2020) Reassessment of *Bournea* Oliver (Gesneriaceae) based on molecular and palynological evidence. PhytoKeys 157(6): 27–41. 10.3897/phytokeys.157.55254 32934446 PMC7467971

[B3] Fedde F (1911) Vermischte neue Diagnosen. *Repertorium specierum novarum regni vegetabilis*. Selbstverlag des Herausgebers, Berlin 10: 64. 10.1002/fedr.19110100109

[B4] Fu LF Li S Xin ZB Wen F Wei YG (2019) The Changes of the Chinese Names and Scientific Names of Gesneriaceae in China between Wang’s and Weber’s Classifications for Gesneriaceae. Guangxi Sciences 26(1): 118–131.

[B5] GRC (2023) [continuously updated] Gesneriaceae Resource Centre. Royal Botanic Garden Edinburgh. https://padme.rbge.org.uk/grc/ [Retrieved/Accessed: 23 Oct 2023]

[B6] Léveillé H (1911a) Decades plantarum novarum. LIX–LXX. *Repertorium specierum novarum regni vegetabilis*. Selbstverlag des Herausgebers, Berlin 9: 447. 10.1002/fedr.19110092706

[B7] Léveillé H (1911b) Decades plantarum novarum. LIV–LVIII. *Repertorium specierum novarum regni vegetabilis*. Selbstverlag des Herausgebers, Berlin 9: 329. 10.1002/fedr.19110091909

[B8] Li HW (1982) Two new genera and one little known genus of Gesneriaceae from Yunnan. Acta Botanica Yunnanica 4(3): 241–247.

[B9] Li ZY Wang YZ (2005) Plants of Gesneriaceae in China. Henan Science and Technology Publishing House, Zhengzhou, 340–416.

[B10] Lv ZY Yusupov Z Zhang DG Zhang YZ Zhang XS Lin N Tojibaev K Sun H Deng T (2022) *Oreocharisxieyongii*, an unusual new species of Gesneriaceae from western Hunan, China. Plant Diversity 44(2): 222–230. 10.1016/j.pld.2021.11.008 35505983 PMC9043400

[B11] Möller M Middleton D Nishii K Wei YG Sontag S Weber A (2011) A new delineation for *Oreocharis* incorporating an additional ten genera of Chinese Gesneriaceae. Phytotaxa 23(1): 1–36. 10.11646/phytotaxa.23.1.1

[B12] Pan KY (1987) Taxonomy of the genus *Oreocharis* (Gesneriaceae). Acta Phytotaxonomica Sinica 25(4): 264–293.

[B13] Turland NJ Wiersema JH Barrie FR Greuter W Hawksworth DL Herendeen PS Knapp S Kusber WH Li DZ Marhold K May TW McNeill J Monro AM Prado J Price MJ Smith GF (2018) International Code of Nomenclature for algae, fungi, and plants (Shenzhen Code) adopted by the Nineteenth International Botanical Congress Shenzhen, China, July 2017. Regnum Vegetabile 159. Koeltz Botanical Books, Glashütten. 10.12705/Code.2018

[B14] Wang WT Pan KY Zhang ZY Li ZY Tao DD Yin WC (1990) Gesneriaceae. In: Wang WT (Ed.) Flora Reipublicae Popularis Sinicae.Vol. 69. Science Press, Beijing, 125–171.

[B15] Wang WT Pan KY Li ZY Weitzman AL Skog LE (1998) Gesneriaceae. In: Wu ZY Raven PH (Eds) Flora of China Vol.18. Science Press, Beijing, and Missouri Botanical Garden Press, St. Louis, 251–261.

[B16] Wang YZ Mao RB Liu Y Li JM Dong Y Li ZY Smith JF (2011) Phylogenetic reconstruction of *Chirita* and allies (Gesneriaceae) with taxonomic treatments. Journal of Systematics and Evolution 49(1): 50–64. 10.1111/j.1759-6831.2010.00113.x

[B17] Weber A Clark JL Möller M (2013) A New Formal Classification of Gesneriaceae. Selbyana 31(2): 68–94.

[B18] Wei YG Wen F Möller M Monro A Zhang Q Gao Q Mou HF Zhong SH Cui C (2010) Gesneriaceae of South China. Guangxi Science & Technology Publishing House, Nanning, 104–675.

[B19] Zhang YM Guo SW Chen WH Shui YM (2019) Rediscovery and conformation of *Oreocharisrhytidophylla* (Gesneriaceae) with a supplementary description of flowers. Guihaia 39(5): 569–573.

